# An in-depth understanding of stakeholders’ experiences about their participation in the co-production of ‘Maze Out’: a serious game for the treatment of eating disorders

**DOI:** 10.1186/s40337-024-01136-3

**Published:** 2024-11-14

**Authors:** Maria Mercedes Guala, Aida Bikic, Kim Bul, David Clinton, Anette Søgaard Nielsen, Helene Nygaard Nielsen, Martina Scarpelli, Luciana Schneider, Regina Christiansen

**Affiliations:** 1 Psychiatric Research Unit, Institute of Clinical Research, University of Southern Denmark, Psychiatric Hospital, J.B. Winsløws vej 18, Odense, Region South Denmark 5000 Denmark; 2 Research Unit Child and Adolescents Psychiatry, Department of Clinical Research, University of Southern Denmark, Odense, Denmark; 3 Research Centre for Intelligent Healthcare, Coventry University, Coventry, UK; 4 Centre for Eating Disorders Innovation (CEDI), Department of Medical Epidemiology and Biostatistics (MEB), Karolinska Institute, Stockholm, Sweden; 5 Centre for Animation, Visualization and Digital Storytelling (CAV), The Animation Workshop, VIA University College, Viborg, Denmark; 6 Faculty of Psychology, National University of Cordoba, Cordoba, Argentina

## Abstract

**Background:**

Co-production is increasingly used in mental health research and clinical settings. Maze Out is a digital game co-produced by clinicians, patients with eating disorders (EDs), an art director with lived experience in EDs, and a game-developing company. Maze Out is based on everyday challenges when suffering from EDs and is currently being evaluated as a supplement tool in EDs treatment. Several studies on co-producing mental health interventions focus on design and effectiveness, but the experiences of those involved in the co-production process remain unexplored. An in-depth exploration of stakeholders’ experiences offers valuable insights into the impact of co-production on different groups and generates crucial knowledge for successful implementation.

**Objectives:**

This study evaluated and explored the co-production process and the meaning that EDs patients, clinicians, and game designers attributed to their participation in the co-production of Maze Out. The objectives were to determine (1) how stakeholders experienced their collaboration in the co-production of Maze Out.; and (2) to what extent the stakeholders involved in developing Maze Out followed Cahn’s principles of equality, diversity, accessibility, and reciprocity.

**Methods:**

Five stakeholders (two patients, two clinicians, and a game designer) who participated in the co-production completed semi-structured interviews. Two patients and one clinician’s diaries supplemented the interviews. Reflexive thematic analysis was used to interpret the data.

**Results:**

The results of this study highlight the importance of building a common language between clinicians, patients, and other professionals involved in developing new forms of treatment and interventions. A recommendation for researchers and clinicians to implement co-production in the future is that Cahn’s principles: equality, reciprocity, accessibility, and diversity, serve as a strong foundation for successful co-production. In this study, three and partially one of the four Cahn’s principles about co-production were identified: equality, reciprocity, and accessibility. When applied in an ED context, these principles provided stakeholders with valuable insights, enriching practice-based knowledge, using the knowledge applicable to clinical practice, and demonstrating their crucial role in fostering effective co-production processes.

**Supplementary Information:**

The online version contains supplementary material available at 10.1186/s40337-024-01136-3.

## Abstract (plain language summary)

This study explored the co-production of Maze Out, a digital game developed as a supplementary tool for eating disorder treatment. Maze Out was co-created by eating disorder patients, clinicians, a game development company, and a designer with lived experience of eating disorders. This study explored how these stakeholders experienced the collaboration and whether they perceived the process to reflect principles like equality, diversity, accessibility, and reciprocity. We interviewed all stakeholders who participated in the co-production process. Two patients and one clinician’s diaries supplemented the interviews. In this study, three fundamental principles—equality, reciprocity, and accessibility—were central. These principles helped participants gain valuable insights and improve their practical and clinical knowledge about eating disorders. The findings highlight the importance of building a shared understanding among clinicians, patients, and other professionals involved in creating new treatments. Researchers and clinicians are recommended to implement Cahn’s principles to support successful collaboration in the future.

**Supplementary Information:**

The online version contains supplementary material available at 10.1186/s40337-024-01136-3.

## Background

There is an increasing recognition of the importance of involving multiple stakeholders in research and health care. The involvement of patients, relatives, clinicians, and researchers has been shown to enhance the quality and relevance of clinical research in mental health and substance abuse, though not yet in eating disorders [1–3]. In a clinical setting, the involvement of multiple stakeholders (a) helps resolve issues that both patients and clinicians are most concerned about, (b) measures treatment effects that are relevant to patients, and (c) increases treatment adherence overall [4–8]. Furthermore, it is recognized that user participation is crucial for the successful development and adoption of new treatment technologies [8–11].

The degree of stakeholder involvement may vary depending on the research context, the project’s specific phase, and the involvement’s aim. Stakeholders may, for example, be involved only as advisors on clinical relevance, as co-investigators, or as working in full partnership throughout the entire research process [1, 12–16].

Co-production, a concept initially developed by Elinor Ostrom and further expanded by Edgar Cahn [17], involves engaging multiple stakeholders in development processes. Cahn has outlined four key principles for operationalizing co-production for it to be successful:

- Reciprocity: all participants should feel valued and be able to contribute to their expectations while receiving benefits in return.
- Equality: no group or person is more important than any other. Each participant has an equal opportunity to contribute.
- Accessibility: everyone has the same opportunity to be involved in the co-production activities in a way that is suitable for them.
- Diversity: participants from diverse backgrounds must be included.

Co-production has different definitions depending on the discipline in which the work is conducted, what is being produced, by whom, and for what purpose [4, 18–20]. The approach of the present study is based on a definition of co-production, a collaborative model where all stakeholders are considered equally important in producing knowledge and interventions. It is further based on the premise that people’s lived experience is a valuable source of knowledge [4, 10]. Co-production is more than consultation and can also include the design, validation, dissemination, and implementation of health interventions [12, 13].

Recently, involving multiple stakeholders and co-production as a method for developing interventions has gained interest within wider mental health settings [21–25]. A recent systematic review by Brotherdale and colleagues (2024) on co-producing digital mental health interventions revealed significant variation in approaches, concluding that future research should focus on understanding stakeholders’ perspectives on co-production [26].

Eating disorders (EDs) comprise severe mental disorders characterized by comprehensive and persistent disturbance in eating behaviors, body image, and associated distressing thoughts and emotions [27]. EDs often arise in adolescence and young adulthood and are more prevalent in women. About 1.4% of women and 0.2% of men experience anorexia nervosa during their lifetime; 1.9% of women and 0.6% of men are affected by bulimia nervosa, while 2.8% of women and 1.0% of men develop binge eating disorder [28]. Although extensive research in the area of EDs has been conducted, the current treatment options prove insufficient, with an overall recovery rate for patients with EDs of 46% [29].

The development and use of digital interventions in EDs have become increasingly common, resulting in a broad range of self-help interventions and treatment tools [30, 31]. Smartphone-based interventions have shown the potential to be effective tools in the treatment and prevention of EDs [32–34]. Digital interventions are accessible, offer the possibility for anonymous access, and meet users’ needs regarding where and when to use them. For patients with EDs, digital interventions provide the possibility to overcome help-seeking barriers such as stigma and shame [35–38]. From a public service perspective, many people can access digital tools regardless of geographical location [35, 39, 40].

Since treatment adherence is a challenge when treating eating disorders, the field of digital mental health is increasingly focusing on developing strategies to improve retention rates and end-user engagement [36, 41]. There is a consensus that enhancing retention, engagement, and symptom improvement necessitates designing digital interventions that meet the end user’s needs [36, 41–43].

Involving multiple stakeholders in designing digital tools for EDs is still the exception rather than the rule. Still, it is becoming more common through approaches like user-centered design, design thinking, and co-production [44–46]. In the present study we describe the development of Maze Out, a serious game coproduced at the Psychiatric Hospital in the Region of Southern Denmark from January to December 2020. Maze Out was developed in close collaboration with four patients with different EDs diagnoses, clinicians with expertise in the field of EDs, an art director, and a game company [46, 47]. The effectiveness of Maze Out in improving treatment outcomes as an add-on to treatment as usual is currently being evaluated through a mixed-method randomized controlled trial (RCT) [47]. In this RCT, the game is intended to be used alongside and asynchronously with general treatment for 15 weeks. Participants will be asked to play at least once a week and can decide how long each session lasts [47].

The rationale behind developing Maze Out was to provide an accessible intervention as an add-on to treatment as usual (which often consists of psychological interventions, nutrition advice, and sometimes medication) [48, 49]. The game is designed to be accessible, enjoyable, and relevant to patients’ daily life issues, as well as their EDs problems and daily life issues, thereby enhancing motivation for treatment [41, 50]. Maze Out is theoretically based on a mentalization approach to EDs treatment. It aligns with current EDs treatment principles, incorporating elements of goal setting, analysis of EDs behaviors, problem-solving, and mentalization exercises [32, 51].

The theoretical foundation of Maze Out is based on a combination of the clinicians’ experiences working in the field of EDs, patient experiences, and research findings on how EDs affect patients’ lives [52, 53]. Impaired insight is considered a common feature of EDs, making it difficult for some patients to recognize and identify where to pinpoint their efforts to make significant life changes [54–56]. Based on the authors’ clinical observations (MG, HN) and as described in the literature, some patients suffering from EDs seem to better understand their inner processes during treatment. This often results in the development of a language for describing how EDs symptoms “infiltrate” patients’ lives [57–62]. The involvement of these patients in developing supplementary treatment tools is crucial, as it enables a holistic understanding of the nature of EDs and facilitates a needs-based approach to treatment [4].

Co-production is a complex process where several different approaches and dimensions can be analyzed. In general, studies on co-producing mental health interventions tend to focus on design and effect, while the experiences of the involved participants themselves remain largely unexplored [63]. However, an in-depth examination of stakeholders’ experiences may offer valuable insights into how co-production impacts the various stakeholder groups involved and may generate crucial knowledge on implementing co-production processes effectively. This qualitative study aimed to evaluate and explore the co-production process, specifically focusing on the perspectives of ED patients, clinicians, and game designers and the meaning they attributed to their participation in developing the serious game Maze Out.

Initially, we decided to investigate how the stakeholders experienced their participation in the co-production of Maze Out. However, it became evident that an additional dimension was needed. As a result, we introduced a second objective: to examine to what extent the co-production process adhered to Cahn’s principles of equality, reciprocity, accessibility, and diversity. These principles provide a framework for evaluating whether the process fostered meaningful collaboration and inclusivity, ensuring that all stakeholders contributed equally and their diverse perspectives were valued.

By applying Cahn’s principles, the analysis evaluated stakeholder experiences and provided a measure of how well the co-production process was implemented. This approach may serve as a valuable guide for future projects, ensuring that co-production processes are equitable and fully operationalized.

## Methods

### Maze Out’s contents

The co-production process resulted in Maze Out: a serious game to be played on a tablet or smartphone, constructed as a labyrinth, a maze [46, 64]. Maze Out is built around a narrative in which the player finds themselves caught in a dream. In this dream, the player is trapped in a maze and can only escape by solving a series of missions, which allows the player to get closer to the exit. The missions are related to ordinary life scenarios linked to personal decisions in which the player needs to decide between two possible options involving choices of actions to be taken or emotions experienced in given circumstances, most related to socializing. There are no right or wrong decisions, and after the player chooses one option, the app shows a validation message related to the feeling that the decision entails. For instance, one of the scenarios of the mission called *“Say what you feel”* presents a situation in which the player cancels plans with a friend and has to choose one of the two options representing their feelings, namely (1) having a bad conscience or (2) feeling proud because of having prioritized their own needs (see Table 1). These choices are associated with complex psychological processes, which the game facilitates identification and approach. This is one of the examples through which the mentalization approach is delivered [51, 65].

**Table 1:**
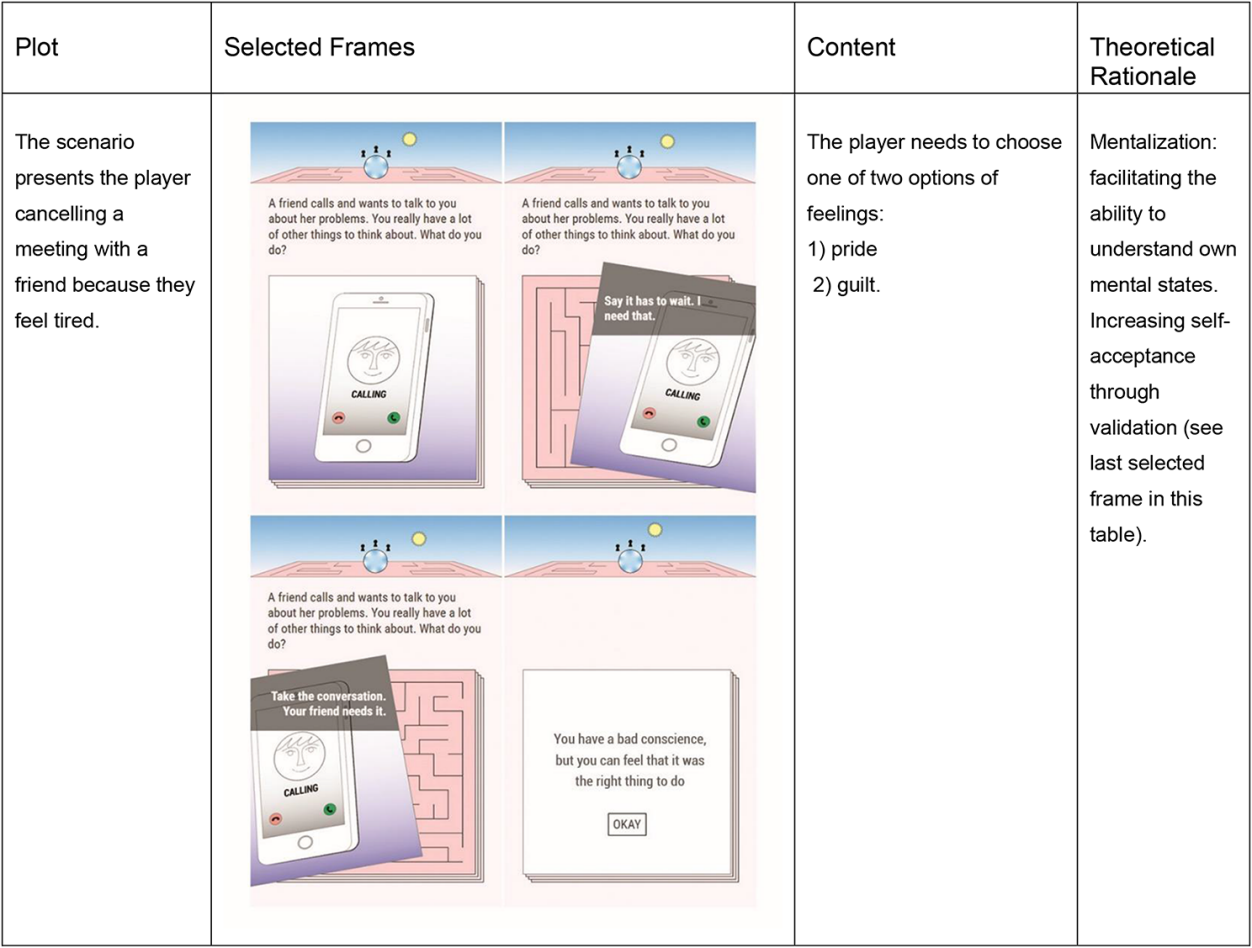
Selected scenario from the mission: “Say what you feel”

**Fig. 1:**
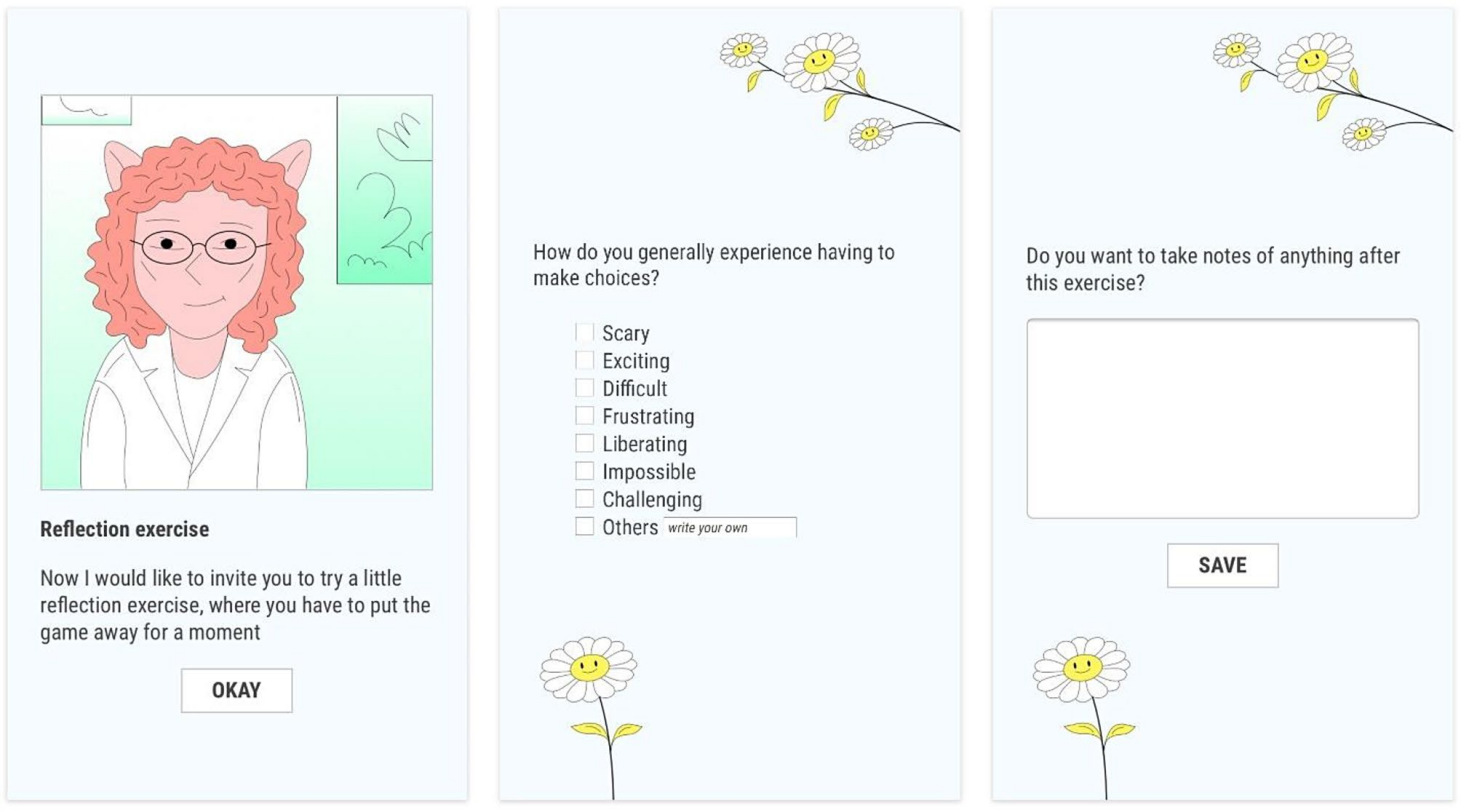
Frames of the reflection activity of the mission: “Say what you feel”

At the end of each mission, there is a reflection activity in which a therapist character in the game encourages the player to identify their feelings or reactions (see Fig. 1). The information is only accessible by the player, who can email it to themselves and eventually share it with their real-life therapist if they wish to. Additionally, some of these sections include an interphase with an option to freely write notes about the feelings experienced.

The player has to go through five portals consisting of a total of 31 missions before the player finds the way out. The missions reflect themes such as food and exercise but also feelings, relationships, and communication. In addition to completing the missions, players are introduced to reflection exercises, inviting them to consider the challenges they meet and to help them relate the game’s content to their everyday lives. During the game, challenges become increasingly demanding and involve complex psychological processes such as setting boundaries, making decisions autonomously, or managing emotions.

A key aspect of the game is its ability to immerse players in a fictional reality while simultaneously prompting them to reflect on their own real-world experiences [47]. In Maze Out, techniques inherent to the language of animation are used, incorporating surreal elements such as talking animals and other unrealistic characters into the game. (see the characterization of the therapist in Fig. 1). The game’s animation facilitates the suspension of disbelief, resulting in the players experiencing fantasy as reality [66, 67]. This may prompt players to freely explore sensitive topics related to ED, which otherwise would be stressful to approach and, therefore, difficult to address.

This aspect of play enhances the game experience and facilitates deeper engagement with the underlying themes of the narrative. The player can experiment with different choices and outcomes, gaining insights into their own experiences while also empathizing with the struggles of the characters they encounter as they explore life.

### Stakeholders involved in the co-production of Maze Out

Five patients diagnosed with EDs, three clinicians from the Psychiatric Hospital in the Region of Southern Denmark, three game designers from Copenhagen Game Lab, and an art designer with lived experiences of EDs were invited to participate in the co-production of Maze Out. Purposive sampling was used to select the stakeholders. The purposeful sampling strategy aimed to identify participants interested in the project as a starting point. Among these interested patients, those determined by their clinicians to have insight into EDs were chosen. All five patients were known by the clinicians participating in this study; four were receiving outpatient treatment, and one had just completed treatment at the time of recruitment to the project. Diversity in terms of socio-economic background, age, gender, and life situation was prioritized to the extent that was possible (Appendix 1).

One patient dropped out of the study for personal reasons before the first workshop. All other participants took part throughout the entire process.

### The co-production process

The co-production team specifically worked on the following tasks: (1) defining the set-up and the layout of the game, (2) describing situations from everyday life that challenge people with EDs, and (3) creating reflection exercises based on those daily life situations.

All stakeholders involved in the process of co-production took up the roles of either producer or evaluator of a given task. The “producer” and “evaluator” positions were used for triangulation when the stakeholders shifted from one position to the other depending on the topic focus. The patients took on the producer role when they provided information about their lived experiences of EDs or how they encountered everyday challenges, and they took on the receiver role when they played the game and gave usability feedback.

The clinicians played a producer role when they developed the reflection tasks and an evaluator role when they evaluated the game as a potential support tool for treatment.

The art director of the game had a twofold position: a lived experience perspective and professional knowledge about game art and design, which allowed both to be integrated in the layout of Maze Out.

The game designers had an implementation role when designing and presenting the game, and a user role when using the information from patients and clinicians to develop the game content. All stakeholders worked together to make decisions on specific tasks, creating a playful universe where EDs were organically integrated with reality. The roles and triangulation of these roles are described in more detail in Table 2.

**Table 2:** Triangulation in the co-production process of Maze Out

Task	Patients (four)	Clinicians (three)	Game designers (three)	Art director
Information about challenges in everyday life when suffering from ED	**Deliver**: Describe situations from everyday life.	**Filter**: Does the given information apply to most ED patients?	**Evaluator**	**Evaluator**
Applicability: mechanics, dynamics and aesthetics	**Evaluator**: Is the produced game content relatable and the mechanic useful?	**Evaluator**: Can the produced game content apply to people suffering from ED?	**Producer**: Use the given information to produce effective game content.	**Producer**
Reflection exercises	**Evaluator**	**Deliver**	**Filter**	**-**
Visual style	**Evaluator**	**Evaluator**	**Evaluator**	**Deliver**

**Fig. 2:**
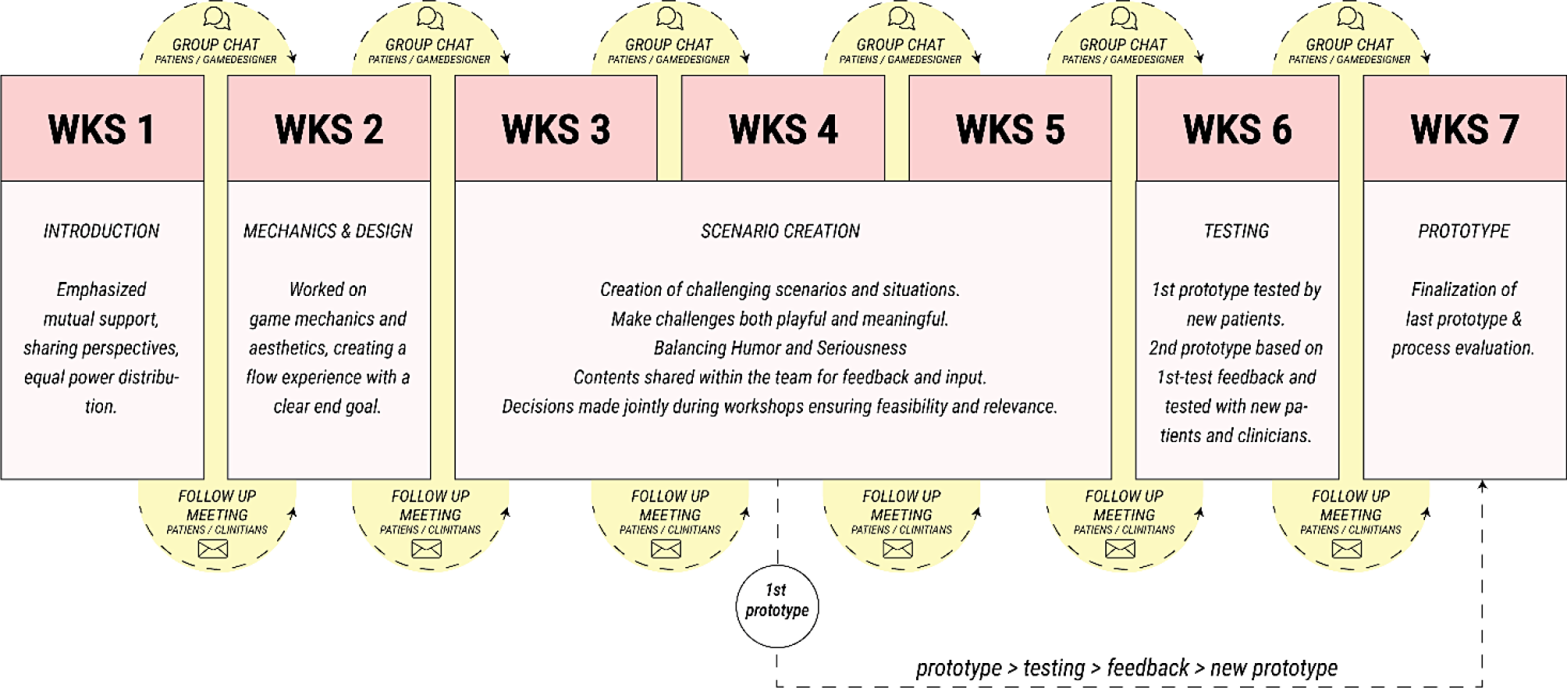
Co-production process of Maze Out

The co-production process included seven workshops of four hours each. Regular mail correspondences and discussions on a private chat forum were used between the workshops, with a focus on minor amendments after each workshop, creating a dynamic and circular pattern of iterations (Fig. 2).

A follow-up meeting between patients and clinicians was held after each workshop to give the patients a place to talk about the process and any difficult situation that could have evolved during the workshops. After the fourth workshop, the first prototype of Maze Out was ready to be tested by a group of 19 patients diagnosed with EDs who had not participated in the game development. The testing of the prototype was conducted through a meeting that was facilitated by a game designer and a patient from the co-production team. Afterward, a second prototype was developed,, implementing the feedback on the first prototype. The second prototype was tested with new patients and clinicians in the last workshop, where the feedback was used to improve the game’s final version.

The COVID-19 pandemic challenged the co-production process to some extent. Although all workshops could be conducted face-to-face, participants had to wear facemasks during the last four workshops and socially distance themselves. The follow-up meetings with all stakeholders were conducted online as video meetings.

### Data collection

Three tailored interview guides were developed to address the different roles of participants throughout the co-production process. These guides focused on participants’ overall experiences of engaging with the co-production process aimed to uncover whether it provided new insights to EDs and, if so, what kind of insights. Participants were aware of MG’s role as a clinician working with EDs patients and the reasons for conducting the research. They were introduced to co-production practically rather than theoretically, both before and during the co-production process. Prior to the interviews, participants were informed that the goal of the interviews was to explore their experiences with the process; however, the term “co-production” was not explicitly mentioned. Cahn’s principles were also not introduced at any point, as they had not yet been explicitly integrated into Maze Out’s co-production process.

All participants were invited to be interviewed and could choose between individual or focus group interviews, depending on their preferences and availability. Four interviews were conducted: two individual interviews (one with a patient and one with the game designer) and two focus group interviews (one with the other two patients and one with the two clinicians). MS, a research assistant not otherwise involved in the study, conducted the patient interviews, while MG conducted the interviews with the clinicians and the game designer. The interviews were conducted face-to-face with the clinicians, and two out of three patients, with the third patient interviewed by telephone and the game designer, interviewed online. The duration of each individual interview or focus group interview was approximately 60 min. All participants were given the opportunity to comment on and/or correct their transcripts. MG and MS took notes after the interviews, which were incorporated into the first step of the analysis (familiarization), as described in Appendix 2.

Data were collected at the Department of Psychiatry Odense and securely stored on the Odense Patient Data Explorative Network (OPEN) platform. All participants were also encouraged to keep a diary throughout the co-production process, and one clinician and two patients did so. The present study also included their diary entries.

The data also comprised video recordings of all workshops performed during the co-production process. All audio recordings of interviews were transcribed verbatim by two research assistants, MS and CO, with all identifying information removed. The diaries were accessible in analog format.

### Data analysis

The data were analyzed by the core research team (MG, HN, DN), who had previously participated in the co-production process. Both inductive and deductive analysis were conducted, as both are valid approaches in reflexive thematic analysis [68, 69]. Data were analyzed without the use of software, through personal discussions among the researchers. A detailed overview of the analysis process can be found in Appendix 2.

The first objective of the study was to investigate how stakeholders experienced their participation in the co-production of Maze Out. This was addressed using an inductive, data-driven approach. This inductive process involved a data-driven approach, implemented by producing codes that were reflective of data content without any pre-conceived theory or conceptual framework [68, 69]. Transcripts were initially read and discussed by a small group of researchers (MG, HN, and DN). MG and HN independently created codes and themes, which were later jointly discussed in order to reach a consensus. MG, a psychiatrist, and HN, a nurse, both with over ten years of clinical experience in EDs, employed personal and epistemological reflexivity throughout the process. Focused discussions on confirmation bias, with RC, a philosopher specializing in qualitative analysis, acting as a sparring partner, helped refine interpretations using critical reflexivity. A subsequent discussion included AN, who has extensive experience with thematic analysis, along with RC. Both, having different theoretical backgrounds and no prior experience with EDs, enriched the analysis through collaborative reflexivity by offering valuable contrasting perspectives. These themes were then summarized and interpreted, focusing on both their explicit meanings and their broader implications and significance. Participants were given the opportunity to provide feedback on the findings as part of a member check to confirm the credibility of the findings. Throughout this analysis process, the core research group used their own knowledge as a resource for interpreting data [68].

The second objective was to investigate to what extent the co-production process during the development of Maze Out adhered to Cahn’s principles of equality, diversity, accessibility, and reciprocity. For this objective, a deductive approach was employed. The process followed the same steps mentioned previously: data familiarization, an initial discussion between MG and HN, followed by a second discussion including RG and AN. MG and HN developed a set of codes based on Cahn’s principles to guide the coding process and inform the theoretical interpretation of the data. After both analyses were completed, a discussion was held to reach a consensus and finalize the thematic analysis. Themes were summarized and interpreted by focusing both on their explicit meaning and on their implications and broader importance [70].

These two processes, drawing on both the researchers’ understanding and the application of Cahn’s principles, enabled the identification of themes extending beyond Cahn’s principles (e.g., hope). This synergy between the researchers’ interpretations and the established principles guided the overall data analysis.

## Results

The data analysis suggested that the co-production process was underpinned by approaches that match Cahn‘s principles [17]. Three and partially one of Cahn’s four principles about co-production were identified: equality, reciprocity, accessibility, and diversity.

Furthermore, key themes about the process of co-producing Maze Out were identified: (1) hope and new insights, (2) going from misunderstanding to a common language, and (3) togetherness/connectedness. These themes will be elaborated one at a time below.

### The extent the stakeholders followed Cahn’s principles

#### Reciprocity

Cahn’s first principle, reciprocity, was accomplished by allowing each stakeholder to express their thoughts and recognitions concerning the Maze Out game.

From the onset, stakeholders held different positions that may have had an influence on their level of reciprocity. The patients seemed to be considered vulnerable by the clinicians in regard to their diagnoses. At the same time, they were considered experts who were able to share knowledge about living a life with EDs. Being in such a position left the patients with manifold feelings of expansion of their own limits, courage, and desire to show more about themselves. In a focus group interview, one patient noted what may be the essence of successful co-production and reasons for engaging in it:

*“ … I don’t know if I was afraid to say it out loud*,*but at least it pushed my boundaries. And when I am supported both by those who work with eating disorders and others who have also experienced an eating disorder*, *it just gave me a boost. So*, *it gave me strength to tell some other things*, *which were also difficult to open up about.” (D*, *patient)*.

The patient above highlighted feeling a boost of energy despite the discussion about eating disorders being surrounded by a sense of transgression. In an excerpt from a diary, some indications of what such a boost might cover were found:

- *“I have been able to be in my own inadequacy*, *AND THAT IS NEW FOR ME AND HUGE because I have felt accepted and included.” (L*, *patient)*.

The uniqueness of the co-production process was grounded mainly on its reciprocal principles that offered each stakeholder the possibility of both contributing to the project and receiving something in return. For the patients, reciprocity involved being accepted and included and further giving the patients an opportunity to accept their own feelings of inadequacy. The reciprocal principle was also found among clinicians who expressed that they did, in fact, learn from the patients’ experiences during the process of co-production since the patients revealed inner experiences that had been kept to themselves during therapy. One clinician put it out this way:

- *“So I*, *I think I learned a lot from the patients as well*, *I think several of the patients shared something that they might not always have said when they came for treatment*, *so I think that was exciting… ” (I*, *clinician)*.

On a practical level, for the sake of developing a shared understanding and communication with the patients, clinicians engaged with themselves in reciprocity in co-production while at the same time being aware of their lack of interest in games for treatment purposes. Yet, the data suggests that sometimes it is necessary to leave one’s field of knowledge to gain extended knowledge. One of the clinicians emphasizes:

- *“… I think it’s important to try to develop communication and at the same time I have no interest in games…Somehow I haven’t really understood or had enough interest in trying to understand that world*, *nor the videos and especially not games*, *so I thought I would probably benefit from that.”’ (E*, *clinician)*.

#### Equality

Cahn’s second principle was considered particularly important in the present project, since patients diagnosed with an ED may be in a vulnerable position due to their disorder, making them less confident in their abilities to contribute. In their diary, a patient reflected on the mutual conversation to gain mutual knowledge, something the patient recognized as a new way for her to reach understanding. In one of the focus group interviews, a participant described feelings of belonging, both in terms of practical contributions and as being part of a community. The patient expressed it in the following way:

- *“(…) the discussions we’ve had*, *were… I thought were incredibly exciting: to get things from different angles*, *and to turn them over and discuss how we should do things. And I found it exciting to hear how we should approach things from different perspectives. These discussions have also occupied my thoughts a lot.” (L*, *patient)*.

The co-production format appeared to facilitate the generation of new knowledge and insights, even for individuals with extensive relevant experience, by creating a space where everyone could contribute equally. One clinician described this way:

- *“..I think that sometimes*, *when we sort of brainstormed together*, *and then one thought of one thing*, *and the other could sort of carry it on*, *that*, *I couldn’t have come up with that myself*, *because there was also something instructive and something surprising in it when they started talking about such completely everyday situations in detail.” (I*, *clinician).*

Developing a digital, serious game by means of co-production seemed to lead to equality not being just a concept that was talked about, but instead led to establishing a tacit relationship of dependency between the parties. Since co-production in this study addressed a relatively complex process around various aspects such as professionalism, emotions, perceptions, technology, etc., which in turn were brought together in a strict format, it required the participation of everyone included. The game developer expressed this by highlighting the importance not only of belonging to the common construction but also of producing a movement of positions and knowledge:

- *“…it underlines the importance of the process we have had*, *which did not just involve you (clinicians) as experts*, *but also involved the target group. Different*, *that is*, *different people from the target group. There are two things. One is that the things that they suggested for content were things that I had no chance of being able to propose before we had those conversations*, *before we had come up with some suggestions*, *and begin to understand so*, *this is what you’re facing when you*, *when you have this ilness.” (N*, *game developer)*.

#### Accessibility

Cahn’s third principle of accessibility refers to whether co-production, in its true sense, allowed all stakeholders, irrespective of their starting point in the project, to step in and contribute to the game design. The principle emphasizes creating an environment where everyone, irrespective of their illness-related knowledge or level of lack of technical expertise, could engage and perceive their involvement as beneficial to the project. This sense of accessibility is reflected in this patient’s diary entry:

- *“It was really exciting to hear everyone’s ideas for the game and see if it can all be assembled into a coherent and meaningful computer game.” (D*, *patient)*.

The patient’s excitement about seeing everyone’s ideas come together highlights how accessibility was achieved. The process allowed her, despite any technical or illness-related barriers, to actively participate and feel a sense of contribution to the overall project.

Having access to equal possibilities to contribute to game development meant that all stakeholders were given the same opportunities to make suggestions for the game. We identified this as a process in which all participants could contribute when they shared a common interest or possessed specialized knowledge, whether that knowledge was professional, technical, or experiential. One patient explained:

*“But I think we all found our place in it. And we were all just given some possibilities*, *and we had to grab what we wanted.” (L*, *patient)*.

For one of the clinicians, dealing with her role in the co-production process was slightly more complicated. Possibly due to professional knowledge and experience, she struggled to enter the process on an equal footing with other participants. It is conceivable that this clinician had to angle her professionalism in a different way than she was used to and allow the process to develop and gain new insights. At first, she seemed to be confused about what happened in the process, and it took time for her to find agreement between the various elements in the process, including which of the parties had influence at different stages of the process. She described it as follows:

*” And then I became a little doubtful about what forces have an influence on the process here. What is going on? Will it be played for the sake of playing*,*or will there be the dynamism and community that was planned for…” (E*, *clinician)*.

#### Diversity

Cahn’s fourth priniciple was achieved, when it came to securing diversity by means of the representation of patients, clinicians, and game developers. Diversity in gender and types of EDs was not achieved. It proved difficult to find men suffering from EDs who wanted to participate, as there are only a few males seeking treatment for EDs. In terms of diagnosis, one patient who suffered from Binge Eating Disorder (BED) dropped out of the co-production process even before the first meeting, and it wasn’t possible to find a replacement. Two men participated from the game company. The clinicians had different academic backgrounds, and the stakeholders had diverse ethnicities. (Appendix 1)

### Key themes about the process of co-producing Maze Out

#### Hope and new insights

Dealing with EDs, whether as patients, as relatives or as clinicians, is difficult and often associated with a feeling of hopelessness. Coming together to co-produce seems to open a space where those feelings can be accepted and experienced as shared, which gave rise to new insights and hope regarding what the co-produced support tool might contribute to others.

Two patients described in their diaries and afterwards in the interviews what to them was experienced as something of hope. To the patients, hope related to the game and the thoughts they had that the game would be helpful for other patients, and these thoughts gave the patient a feeling of meaningfulness about their own contribution:

- *“I want so much the girls (future players) to be continuously forced into/down into the body and become conscious of it - NOT SO MUCH HEAD*, *MORE ATTENTION TO THE HERE AND NOW.” (L*, *patient)*.
- *The fact that we have to cover such a broad spectrum makes it complex but also exciting at the same time. I’m excited about what’s to come! I feel that I am doing something that makes sense. So right now*, *as I write this*, *I feel hopeful and happy!” (D*, *patient)*.

Clinicians expressed hope in terms of seeing the project develop with enthusiasm and in a way where everyone was heard:

- “The dynamics and interaction in the group is good. F (*F*: game developer) conveys commitment, *overview and belief that the game will be finished and good. The group is more actively participating and gives good feedback and contributes with important questions. Shares the joy that the project can be completed…” (E*, *clinician)*.

The situations and examples that the patients brought into play during the process were crucial in creating insights into how EDs can unfold in everyday life.

- *“I think you can see that when we talked about situations*, *very small things to me were incredibly important to the patients*, *and I mean*, *it was pretty exciting to see… that actually carried over into the game*, *you know*, *in a way where you could allow yourself to think you were like that when you were in this game*, *but at the same time it was also kind of safe.” (N*, *game developer)*.

#### From misunderstanding to common language

Having knowledge defines both a role and vocabulary on how to refer to that knowledge. In the present study, patients would speak from knowledge of lived experience, the clinicians from intellectual and academic knowledge and clinical experiences, and the game designers from knowledge about game mechanics, dynamics, and aesthetics. Therefore, one of the challenges the co-production of the game was creating a space where a common understanding and language could emerge. A patient expressed in her diary and interview the following:

*“ …I thought that at the beginning*, *you could tell that they had to figure out all this eating disorder stuff and get into it and into our world. And I also thought that we should just have time to find out about their world and what could be done. Because I have absolutely no understanding of technology and computers. So*, *it was right there at the beginning where we had to find each other and a common understanding. Because at the beginning*, *it was a bit like they didn’t fully understand us*, *and also*, *I didn’t fully understand the technical aspects. But it seems to me that along the way*, *the more we worked with the game*, *the more there was a common understanding. And we found out how to work together. So*, *it was right*, *it was really good towards the end of the process.” (D*, *patient)*.

For the clinicians, it was particularly important to develop a common language with the game company, as they found that the game designers had a markedly different language at the onset of the project. A clinician described this in the interview:

- *“And then I really think*, *when you think about it*, *like that with the language*, *they showed up with at the very first workshops*, *the game developers*, *and then to where we got to*, *it’s actually quite unique and crazy*, *really.” (I*, *clinician)*.

For the game designer it was challenging to develop a common language but meaningful in terms of developing and improving his ideas. As he expressed it:

- *“…but it was definitely a challenge to explain*, *get the working group to understand what our idea was and conversely take the inputs that the working group came up with on the basis of something half-finished and then add to it and still make the idea better. It was*, *it was a huge challenge also in relation to having a common language. Such a thing as things inside my*,*a map inside my head*, *I know exactly what is in the game*, *but it has taken some time before everyone agreed on what a map actually is.” (N*, *game designer)*.

Here the distinction between aggregating the parts (views, approaches) subsequent to the process and the position of conceiving a collective production from the outset is elaborated.

#### Togetherness/connectedness

When analyzing the data, we became aware of an aspect of co-production that seemed fundamental for the process: the recognition of the other as an active and constructive social subject. Such awareness generated a space of connection in which the parties involved felt part of a common whole.

One patient described it in her diary:

- *Be part of fellowships…. The game development group gives me energy right now… Then you discover that what you yourself thought*, *others thought too*, *and then you refine your language and put it into words… (D*, *patient)*.

Clinicians similarly described how the feeling of being connected developed throughout the process and the results were experienced as a confirmation of a common purpose:

- *“And there was also that…*, *really such a sense of unity that now we have come so far*, or like when they (*they*: game company) came and showed something new and everyone was like “YES, *that is the right way.” (I*, *clinician)*.

## Discussion

The present study used interviews and qualitative diaries to investigate the extent and implications of the co-production process in the development of Maze Out, a serious game for the treatment of EDs. Although the sample size was small, all key stakeholders involved in the development of the game, patients, clinicians, and game designers, were represented. The themes not specifically addressed during the interviews were inductively identified, and these topics appeared crucial for generating clinically significant content for the game. The reflexive thematic analysis then applied a deductive approach to examine the extent to which the co-production process adhered to Cahn’s principles.

Analysis of data suggested that the co-production process of Maze Out took place in a space characterized by mutual respect, where participants created a common language that facilitated the generation of meaningful content for the game, which led to a shared understanding and new insights for all involved. Our findings have important applications for researchers and clinicians who are engaged in the development of treatment tools. Researchers can utilize our results to understand and design better patient-centered studies. At the same time, clinicians can apply our findings to create more tailored and effective treatment strategies, ultimately improving patient outcomes.

The co-production process of Maze Out adhered to three (out of four) of Cahn’s principles throughout, namely equality, reciprocity, and accessibility. These principles provided a foundation from which all stakeholders enriched their understanding of EDs, and in ways they had not experienced before. However, whether or not equality between the stakeholders involved was ensured could be contested when it comes to clinicians. They held the position of being ‘experts’ in the field of EDs and were seen to know more about the patients’ disorders than patients themselves, particularly in terms of optimal and most effective treatment. Clinicians, therefore, had to balance their professional knowledge with openness to new insights gained during co-production to ensure equality.

The findings of the present study underline that a common language is crucial in a co-production process but it can not be taken for granted. People suffering from ED commonly experience or perceive language differently than is intended and have a tendency to express their inner world through the concreteness of the symptoms [51, 71]. Finding a common language probably requires a mentalization process whereby both the concrete and the metaphoric aspects of the symptoms are taken into account [51]. Creating a common language fostered a sense of togetherness, offering a safe space for developing insights within a hermeneutic circle, where each insight built upon and deepened the others.

A common language allowed a greater depth to emerge in the game content. It would have been impossible to achieve these new insights from each stakeholder alone. Therefore the game Maze Out represents new and interdisciplinary knowledge that potentially may allow future users (patients and clinicians) to understand EDs in new ways. This finding highlights the importance of creating a common language between clinicians and patients as a means of understanding the challenges patients face. It also emphasizes the need to find a way to refer to these challenges and their possible solutions that make sense to both parties.

Clinicians reported gaining insights about aspects of EDs that were new to them and acknowledged that this is something they do not have access to during their clinical practice, although all of them had extensive experience of working with EDs. This was an unexpected finding. A possible explanation may be that in clinical practice, there is an unequal distribution of power, as EDs treatment usually involves aspects of treatment that are mandatory (i.e.non-negotiable) [72, 73]. Results of the present study highlight how experiencing equality may allow for new knowledge to emerge, showing the potential of new ways of thinking in clinical contexts. When clinicians and patients interact as equals —where each party’s perspectives and contributions are valued—an environment of mutual respect and collaboration is created. In such an environment, the exchange of ideas can be more open and fluid. Patients can feel empowered to share their personal experiences and challenges that they might otherwise keep to themselves due to shame or fear of being misunderstood. This dynamic can lead to the discovery of new insights that might not emerge in a more hierarchical or unequal interaction. For example, patients might reveal aspects of their condition that were previously overlooked, while clinicians might offer new perspectives or solutions that had not been considered before. When equality is experienced, both parties are more likely to engage in meaningful dialogue, which can challenge existing assumptions and foster innovative thinking.

The community formed through the co-production process, where all parties involved identified with a common whole, was likely a key factor in facilitating a deeper understanding of clinician and patient perspectives of EDs. This could be partly explained by the fact that patients felt secure in being a valued part of a union, the co-production team.

During the present co-production process game designers realized the importance of having an exchange of expertise on product development. The importance of user involvement in serious game design is broadly recognized and recommended as it has shown that this increases the usability and feasibility of the game [74, 75]. Furthermore, in mental health disorders, user involvement in the design process is considered to be a key factor for making a game relevant and effective [76]. Our study shows other aspects of what such an involvement can bring. All stakeholders not only added to the quality of the product that they developed together; they also felt empowered and wiser by participating in the process.

Throughout the co-production process of Maze Out, patients experienced hope, which was realised by the fact that their insight about EDs was considered helpful, not only to themselves but also to others. Co-production empowered them by adding new deepened meaning to their own suffering.

For clinicians, the experience of gaining knowledge about new aspects of ED by participating in co-production suggests that engaging in clinical work only gives a glimpse of the ED universe. Engaging in co-production highlights the importance of preserving a curious “not knowing” stance, underscoring the value of open-minded approach to meeting patients, no matter what the level of clinical expertise is.

The connection space created by the co-production process, where the parties involved identified with a common whole, was likely a decisive factor in overcoming certain barriers to a deeper comprehension of EDs, such as shame. This could enrich hospital practice by encouraging clinicians to actively generate spaces where this sense of togetherness can arise.

### Strengths and limitations

Although our study was limited by a small sample size, all key stakeholders involved in the development: patients, clinicians, and game designers were represented, ensuring a comprehensive view of the co-production process. However, the relatively small number of participants may still affect the depth and variety of perspectives, particularly in terms of capturing a wider range of experiences within different types of EDs. What’s more, the stakeholder group tended to lack diversity, with a preponderance of women and no representation from individuals with BED. This limitation may have influenced the breadth of perspectives captured, particularly regarding gender diversity and the range of lived experiences related to different types of EDs. Including stakeholders of other genders and individuals with BED could have enriched the clinical and experiential perspectives, potentially broadening the content of the game to make it more relatable to a wider range of individuals with EDs. The biases present in the study, such as the epistemological bias of viewing patients as key holders of knowledge and the psychodynamic theoretical bias, may contribute to its limitations.

The study, however, also has considerable strengths. The involvement of experienced researchers without these biases, such as RC and AN, helped mitigate potential problems by providing contrasting perspectives and reducing the risk of biased conclusions. Another strength of this study was the collection of data from all involved parties, including patients and game designers.

## Conclusion

The co-production of Maze Out fully followed three and partially one of Cahn’s four principles: equality, reciprocity, accessibility, and diversity. When these principles were applied in an ED context, they seemed to give stakeholders access to new insights that could enrich knowledge and clinical practice. The results of this study highlight the importance of building a common language between clinicians, patients, and other professionals involved in the development of new forms of treatment and interventions. The use of co-production may generate a feeling of cohesion among stakeholders, allowing for more fruitful future research and clinical practice. The analysis of stakeholders’ experiences in the co-production of Maze Out as a whole seems to indicate that these principles play a decisive role in creating a foundation that allows stakeholders to interact meaningfully.

## Electronic Supplementary Material

Below is the link to the electronic supplementary material.

Supplementary Material 1

Supplementary Material 2

Supplementary Material 3

## Data Availability

Data that support the findings of this study have been deposited in “OPEN - Open Patient data Explorative Network” registration ID: OP_1621.

## References

[1] Hawke LD, Sheikhan NY, Roberts S, McKee S. Research evidence and implementation gaps in the engagement of people with lived experience in mental health and substance use research: a scoping review. Res Involv Engagem. 2023;9(1):1–12. 37170357 10.1186/s40900-023-00442-5 PMC10176886

[2] Johnston JN, Ridgway L, Cary-Barnard S, Allen J, Sanchez-Lafuente CL, Reive B, et al. Patient oriented research in mental health: matching laboratory to life and beyond in Canada. Res Involv Engagem. 2021;7(1):1–11. 33902751 10.1186/s40900-021-00266-1 PMC8074277

[3] Arumugam A, Phillips LR, Moore A, Kumaran SD, Sampath KK, Migliorini F, et al. Patient and public involvement in research: a review of practical resources for young investigators. BMC Rheumatol. 2023;7(1):2. 36895053 10.1186/s41927-023-00327-w PMC9996937

[4] Norton M. Implementing co-production in traditional statutory mental health services. Mental Health Pract. 2024;27(1).

[5] Wiering B, de Boer D, Delnoij D. Patient involvement in the development of patient-reported outcome measures: a scoping review. Health Expect. 2017;20(1):11–23. 26889874 10.1111/hex.12442 PMC5217930

[6] Saesen R, Lejeune S, Quaglio G, Lacombe D, Huys I. Views of European drug development stakeholders on treatment optimization and its potential for use in decision-making. Front Pharmacol. 2020;11:43. 32116718 10.3389/fphar.2020.00043 PMC7015135

[7] Poremski D, Sagayadevan VDO, Wang P, Lum A, Subramaniam M, Ann CS. The impact of stakeholder preferences on service user adherence to treatments for schizophrenia and metabolic comorbidities. PLoS ONE. 2016;11(11):e0166171. 27851771 10.1371/journal.pone.0166171 PMC5112999

[8] Torous J, Nicholas J, Larsen ME, Firth J, Christensen H. Clinical review of user engagement with mental health smartphone apps: evidence, theory and improvements. BMJ Ment Health. 2018;21(3):116–9. 10.1136/eb-2018-102891 PMC10270395 29871870

[9] Kushniruk A, Nøhr C. Participatory design, user involvement and health IT evaluation. Stud Health Technol Inf. 2016;222:139–51. 27198099

[10] Tang T, Lim ME, Mansfield E, McLachlan A, Quan SD. Clinician user involvement in the real world: Designing an electronic tool to improve interprofessional communication and collaboration in a hospital setting. Int J Med Informatics. 2018;110:90–7. 10.1016/j.ijmedinf.2017.11.011 29331258

[11] Wisniewski H, Torous J. Digital navigators to implement smartphone and digital tools in care. Acta Psychiatr Scand. 2020;141(4):350–5. 31930477 10.1111/acps.13149 PMC7928068

[12] Carman KL, Dardess P, Maurer M, Sofaer S, Adams K, Bechtel C, et al. Patient and family engagement: a framework for understanding the elements and developing interventions and policies. Health Aff. 2013;32(2):223–31. 10.1377/hlthaff.2012.1133 23381514

[13] Hardyman W, Daunt KL, Kitchener M. Value co-creation through patient engagement in health care: a micro-level approach and research agenda. Public Manage Rev. 2015;17(1):90–107.

[14] Trevillion K, Stuart R, Ocloo J, Broeckelmann E, Jeffreys S, Jeynes T, et al. Service user perspectives of community mental health services for people with complex emotional needs: a co-produced qualitative interview study. BMC Psychiatry. 2022;22(1):1–18. 35081929 10.1186/s12888-021-03605-4 PMC8791764

[15] Livanou M, Bull M, Manitsa I, Hunt J, Lane R, Heneghan A. Co-producing a complex psychosocial intervention during COVID‐19 with young people transitioning from adolescent secure hospitals to adult services in England: Moving Forward intervention (MFi). Child and Adolescent Mental Health. 2023. 10.1111/camh.12667 37455024

[16] Masterson D, Areskoug Josefsson K, Robert G, Nylander E, Kjellström S. Mapping definitions of co-production and co‐design in health and social care: a systematic scoping review providing lessons for the future. Health Expect. 2022;25(3):902–13. 35322510 10.1111/hex.13470 PMC9122425

[17] Cahn ES. Co-producing justice: The new imperative. UDC L Rev. 2000;5:105.

[18] Pestoff V. Co-production and third sector social services in Europe: Some concepts and evidence. Voluntas. 2012;23:1102–18.

[19] Cepiku D, Giordano F. Co-Production in Developing Countries: Insights from the community health workers experience. Public Manage Rev. 2014;16(3):317–40.

[20] Filipe A, Renedo A, Marston C. The co-production of what? Knowledge, values, and social relations in health care. PLoS Biol. 2017;15(5):e2001403. 28467412 10.1371/journal.pbio.2001403 PMC5414996

[21] Norton M. Implementing co-production in traditional statutory mental health services. Mental Health Pract. 2022;25(5).

[22] Norton MJ. Co-production within child and adolescent mental health: a systematic review. Int J Environ Res Public Health. 2021;18(22):11897. 34831653 10.3390/ijerph182211897 PMC8623106

[23] Horgan A, Manning F, Bocking J, Happell B, Lahti M, Doody R, et al. To be treated as a human’: Using co-production to explore experts by experience involvement in mental health nursing education–The COMMUNE project. Int J Ment Health Nurs. 2018;27(4):1282–91. 29377483 10.1111/inm.12435

[24] Gheduzzi E, Masella C, Segato F. Implementing co-production in mental health organizations. J Mental Health Train Educ Pract. 2019;14(6):480–92.

[25] Faulkner A, Carr S, Gould D, Khisa C, Hafford-Letchfield T, Cohen R, et al. Dignity and respect’: An example of service user leadership and co‐production in mental health research. Health Expect. 2021;24:10–9. 31556244 10.1111/hex.12963 PMC8137502

[26] Brotherdale R, Berry K, Branitsky A, Bucci S. Co-producing digital mental health interventions: A systematic review. Digit Health. 2024;10:20552076241239172. 38665886 10.1177/20552076241239172 PMC11044797

[27] American Psychiatric Association, Association D. AP. Diagnostic and statistical manual of mental disorders: DSM-5. American psychiatric association Washington, DC; 2013.

[28] Treasure J, Hübel C, Himmerich H. The evolving epidemiology and differential etiopathogenesis of eating disorders: implications for prevention and treatment. World Psychiatry. 2022;21(1):147. 35015360 10.1002/wps.20935 PMC8751578

[29] Solmi M, Monaco F, Højlund M, Monteleone AM, Trott M, Firth J, et al. Outcomes in people with eating disorders: a transdiagnostic and disorder-specific systematic review, meta‐analysis and multivariable meta‐regression analysis. World Psychiatry. 2024;23(1):124–38. 38214616 10.1002/wps.21182 PMC10785991

[30] Ahmadiankalati M, Steins-Loeber S, Paslakis G. Review of randomized controlled trials using e-health interventions for patients with eating disorders. Front Psychiatry. 2020;11:568. 32595546 10.3389/fpsyt.2020.00568 PMC7304304

[31] Melioli T, Bauer S, Franko DL, Moessner M, Ozer F, Chabrol H, et al. Reducing eating disorder symptoms and risk factors using the internet: A meta-analytic review. Int J Eat Disord. 2016;49(1):19–31. 26607683 10.1002/eat.22477

[32] Wasil AR, Patel R, Cho JY, Shingleton RM, Weisz JR, DeRubeis RJ. Smartphone apps for eating disorders: A systematic review of evidence-based content and application of user‐adjusted analyses. Int J Eat Disord. 2021;54(5):690–700. 33534176 10.1002/eat.23478

[33] Fairburn CG, Rothwell ER. Apps and eating disorders: A systematic clinical appraisal. Int J Eat Disord. 2015;48(7):1038–46. 25728705 10.1002/eat.22398 PMC4737215

[34] Taylor CB, Graham AK, Flatt RE, Waldherr K, Fitzsimmons-Craft EE. Current state of scientific evidence on Internet-based interventions for the treatment of depression, anxiety, eating disorders and substance abuse: an overview of systematic reviews and meta-analyses. Eur J Pub Health. 2021;31(Supplement1):i3–10. 32918448 10.1093/eurpub/ckz208 PMC8495688

[35] Kazdin AE, Fitzsimmons-Craft EE, Wilfley DE. Addressing critical gaps in the treatment of eating disorders. Int J Eat Disord. 2017;50(3):170–89. 28102908 10.1002/eat.22670 PMC6169314

[36] Linardon J, Shatte A, Messer M, Firth J, Fuller-Tyszkiewicz M. E-mental health interventions for the treatment and prevention of eating disorders: An updated systematic review and meta-analysis. J Consult Clin Psychol. 2020;88(11):994. 32852971 10.1037/ccp0000575

[37] Ali K, Fassnacht DB, Farrer L, Rieger E, Feldhege J, Moessner M, et al. What prevents young adults from seeking help? Barriers toward help-seeking for eating disorder symptomatology. Int J Eat Disord. 2020;53(6):894–906. 32239776 10.1002/eat.23266

[38] Nicula M, Pellegrini D, Grennan L, Bhatnagar N, McVey G, Couturier J. Help-seeking attitudes and behaviours among youth with eating disorders: a scoping review. J Eat Disorders. 2022;10(1):21. 10.1186/s40337-022-00543-8 PMC8845232 35164872

[39] Bauer S, Moessner M. Harnessing the power of technology for the treatment and prevention of eating disorders. Int J Eat Disord. 2013;46(5):508–15. 23658102 10.1002/eat.22109

[40] Ali K, Farrer L, Fassnacht DB, Gulliver A, Bauer S, Griffiths KM. Perceived barriers and facilitators towards help-seeking for eating disorders: A systematic review. Int J Eat Disord. 2017;50(1):9–21. 27526643 10.1002/eat.22598

[41] Barakat S, Maguire S, Smith KE, Mason TB, Crosby RD, Touyz S. Evaluating the role of digital intervention design in treatment outcomes and adherence to eTherapy programs for eating disorders: A systematic review and meta-analysis. Int J Eat Disord. 2019;52(10):1077–94. 31328815 10.1002/eat.23131

[42] Tregarthen J, Kim JP, Sadeh-Sharvit S, Neri E, Welch H, Lock J. Comparing a tailored self-help mobile app with a standard self-monitoring app for the treatment of eating disorder symptoms: Randomized controlled trial. JMIR Mental Health. 2019;6(11):e14972. 31750837 10.2196/14972 PMC6895866

[43] Yardley L, Morrison L, Bradbury K, Muller I. The person-based approach to intervention development: application to digital health-related behavior change interventions. J Med Internet Res. 2015;17(1):e4055. 10.2196/jmir.4055 PMC4327440 25639757

[44] Graham AK, Neubert SW, Chang A, Liu J, Fu E, Green EA, et al. Applying user-centered design methods to understand users’ day-to-day experiences can inform a mobile intervention for binge eating and weight management. Front Digit Health. 2021;3:651749. 34713124 10.3389/fdgth.2021.651749 PMC8521863

[45] Jarman HK, McLean SA, Rodgers R, Fuller-Tyszkiewicz M, Paxton S, O’Gorman B, et al. Informing mHealth and web-based eating disorder interventions: combining lived experience perspectives with design thinking approaches. JMIR Formative Res. 2022;6(10):e38387. 10.2196/38387 PMC9664336 36315225

[46] Guala MM, Bul K, Skårderud F, Søgaard Nielsen A. A serious game for patients with eating disorders (Maze Out): pilot user experience and acceptance study. JMIR Formative Res. 2023;7:e40594. 10.2196/40594 PMC9919769 36705956

[47] Guala MM, Bikic A, Bul K, Clinton D, Mejdal A, Nielsen HN, et al. Maze Out: a study protocol for a randomised controlled trial using a mix methods approach exploring the potential and examining the effectiveness of a serious game in the treatment of eating disorders. J Eat Disorders. 2024;12(1):35. 10.1186/s40337-024-00985-2 PMC10908122 38429839

[48] Wilson GT, Shafran R. Eating disorders guidelines from NICE. Lancet. 2005;365(9453):79–81. 15639682 10.1016/S0140-6736(04)17669-1

[49] Hilbert A, Hoek HW, Schmidt R. Evidence-based clinical guidelines for eating disorders: international comparison. Curr Opin Psychiatry. 2017;30(6):423–37. 28777107 10.1097/YCO.0000000000000360 PMC5690314

[50] Graham AK, Kosmas JA, Massion TA. Designing digital interventions for eating disorders. Curr psychiatry Rep. 2023;25(4):125–38. 36928767 10.1007/s11920-023-01415-x

[51] Robinson P, Skårderud F, Sommerfeldt B. Mentalization-based Treatments for Eating Disorders: Springer; 2018.

[52] Roncero M, Belloch A, Perpiñá C, Treasure J. Ego-syntonicity and ego-dystonicity of eating-related intrusive thoughts in patients with eating disorders. Psychiatry Res. 2013;208(1):67–73. 23541243 10.1016/j.psychres.2013.01.006

[53] Bulik CM, Kendler KS. I Am What I (Don’t) Eat: establishing an identity independent of an eating disorder. Am J Psychiatry. 2000;157(11):1755–60. 11058469 10.1176/appi.ajp.157.11.1755

[54] Casasnovas C, Fernández-Aranda F, Granero R, Krug I, Jiménez‐Murcia S, Bulik C, et al. Motivation to change in eating disorders: clinical and therapeutic implications. Eur Eat Disorders Review: Prof J Eat Disorders Association. 2007;15(6):449–56. 10.1002/erv.780 17960774

[55] Roncero M, Perpiñá C, Belloch A. Ego-syntonicity and eating disorders. New developments in anorexia nervosa research. 2014:107 – 26.

[56] Konstantakopoulos G, Tchanturia K, Surguladze S, David A. Insight in eating disorders: clinical and cognitive correlates. Psychol Med. 2011;41(9):1951–61. 21211101 10.1017/S0033291710002539

[57] Noordenbos G. Important factors in the process of recovery according to patients with anorexia nervosa. The course of eating disorders: Long-term follow-up studies of anorexia and bulimia nervosa. Springer; 1992. pp. 304–22.

[58] Vitousek K, Watson S, Wilson GT. Enhancing motivation for change in treatment-resistant eating disorders. Clin Psychol Rev. 1998;18(4):391–420. 9638355 10.1016/s0272-7358(98)00012-9

[59] Scanferla E, Pachoud P, Gorwood B, E CPGB-JEDADCGEHFJDLALVLZPIRARLS. Experiencing eight psychotherapy approaches devoted to eating disorders in a single-day workshop increases insight and motivation to engage in care: a pilot study. Eat Weight Disorders-Studies Anorexia Bulimia Obes. 2022;27(6):2213–22. 10.1007/s40519-022-01365-y 35133642

[60] Lamoureux MM, Bottorff JL. Becoming the real me: Recovering from anorexia nervosa. Health Care Women Int. 2005;26(2):170–88. 15804915 10.1080/07399330590905602

[61] Reindl SM. Sensing the self: Women’s recovery from bulimia. Harvard University Press; 2001.

[62] Weaver K, Wuest J, Ciliska D. Understanding women’s journey of recovering from anorexia nervosa. Qual Health Res. 2005;15(2):188–206. 15611203 10.1177/1049732304270819

[63] Illarregi ER. Co-Design as Healing: Exploring the Experiences of Participants Facing Mental Health Problems. Open University (United Kingdom); 2021.

[64] Loh CS, Sheng Y, Ifenthaler D. Serious games analytics: Theoretical framework. Serious games analytics: Methodologies for performance measurement, assessment, and improvement. 2015:3–29.

[65] Bateman AW, Fonagy P. Handbook of mentalizing in mental health practice. American Psychiatric Pub; 2019.

[66] Böcking S. Suspension of disbelief. The international encyclopedia of communication. 2008.

[67] Coleridge ST. Biographia Literaria: Samuel Taylor Coleridge. Oxford; 1985.

[68] Braun V, Clarke V. Using thematic analysis in psychology. Qualitative Res Psychol. 2006;3(2):77–101.

[69] Byrne D. A worked example of Braun and Clarke’s approach to reflexive thematic analysis. Qual Quant. 2022;56(3):1391–412.

[70] Patton MQ. Qualitative evaluation and research methods. SAGE Publications, inc; 1990.

[71] Skårderud F. Eating one’s words, part II: The embodied mind and reflective function in anorexia nervosa—theory. Eur Eat Disorders Review: Prof J Eat Disorders Association. 2007;15(4):243–52. 10.1002/erv.778 17676695

[72] Health NCCfM. Eating disorders: core interventions in the treatment and management of anorexia nervosa, bulimia nervosa and related eating disorders. 2004. 23346610

[73] Geller J, Srikameswaran S. Treatment non-negotiables: Why we need them and how to make them work. Eur Eat Disorders Review: Prof J Eat Disorders Association. 2006;14(4):212–7.

[74] Fanfarelli JR, McDaniel R, Crossley C. Adapting UX to the design of healthcare games and applications. Entertainment Comput. 2018;28:21–31.

[75] Verschueren S, Buffel C, Vander Stichele G. Developing theory-driven, evidence-based serious games for health: framework based on research community insights. JMIR serious games. 2019;7(2):e11565. 31045496 10.2196/11565 PMC6521217

[76] Gómez-Cambronero Á, Mann A-L, Mira A, Doherty G, Casteleyn S. Smartphone-based serious games for mental health: a scoping review. Multimedia Tools Appl. 2024:1–48. 10.1007/s11042-024-18971-w PMC11564251 39553422

