# Supplementary material for: An in-depth understanding of stakeholders’ experiences about their participation in the co-production of ‘Maze Out’: a serious game for the treatment of eating disorders
Source: J Eat Disord. 2024 Nov 14;12:178. doi: 10.1186/s40337-024-01136-3 (PMC11566361; doi:10.1186/s40337-024-01136-3)
Supplement: Supplementary file 1 — Supplementary Material 1 [file 40337_2024_1136_MOESM1_ESM.docx]

Appendix 1: participants

| Patients | 4 |
| --- | --- |
| Age | 19, 25, 28, 35 |
| *ED diagnosis:*  Anorexia nervosa  Bulimia nervosa  Eating disorder not otherwise specified | 2  1  1 |
| *Length of ED treatment:*  In the middle of treatment  At the end of the treatment’  Over 5 years  Finished treatment | 1  1  1  1 |
| *Academic background:*  High school  Shorter education  Medium-length higher education | 2  1  1 |

| Clinicians | 3 |
| --- | --- |
| Age | 35, 48, 65 |
| *Academic background*  Social worker  Nurse  Psychiatrist | 1  1  1 |
| *Gender*  Female | 3 |

| Game develompment | 3 |
| --- | --- |
| Age | 36, 43, 47, 52 |
| *Academic background*  Game & education  Art | 3  1 |
| *Nationality*  Danish International | 3  1 |
| *Gender*  Female Male | 2 2 |
