# Supplementary material for: An in-depth understanding of stakeholders’ experiences about their participation in the co-production of ‘Maze Out’: a serious game for the treatment of eating disorders
Source: J Eat Disord. 2024 Nov 14;12:178. doi: 10.1186/s40337-024-01136-3 (PMC11566361; doi:10.1186/s40337-024-01136-3)
Supplement: Supplementary file 2 — Supplementary Material 2 [file 40337_2024_1136_MOESM2_ESM.pdf]

Appendix 2

Tables outlining the Reflective Analysis Process for Gaining an In-depth Understanding of Stakeholders' Experiences in the Co-production of 'Maze Out'

(Adapted from Braun & Clarke, 2019, Reflecting on Reflexive Thematic Analysis, Qualitative Research in Sport, Exercise and Health, 11(4), 589-597)

Table 1. Process during the Inductive Analysis to Address the First Objective: How Stakeholders Experienced Their Collaboration in the Co-production of Maze Out

Step	Description
**1. Data familiarization**	MG, HN, and DN read the interview transcripts, field notes, and diaries, noting their initial impressions and observations.
**2. Initial coding**	MG and HN conducted the initial coding of the transcripts and diaries, identifying key themes: 1) From misunderstanding to shared understanding, 2) Distributed power, 3) Knowledge synergy, and 4) Building a shared universe, characterized by elements such as humor and meaning, as well as emotions like hope and enthusiasm.
**3. Developing themes**	MG and HN refined the initial codes and themes through further analysis. HN applied personal and epistemological reflexivity, reflecting on their clinical experience and the underlying assumptions that could influence the interpretation of the data. This process helped ensure that the themes represented the participants' experiences and not biased by the researchers' preconceived notions.
**4. Reviewing themes**	In this step, RC, a philosopher specializing in qualitative analysis, joined MG and HN to refine the themes further. Through discussions using critical reflexivity, the team critically examined how their assumptions and biases might influence the analysis. RC's participation provided an external perspective, helping to challenge and deepen the understanding of the themes, ensuring they accurately reflected the data rather than confirming pre-existing expectations. This collaborative process helped to solidify the themes and address potential biases.
**5. Refining and naming themes**	MG developed initial proposals for the finalized themes, which were then discussed with HN and RC. AN, with extensive experience in thematic analysis, was also brought in for this step. The collaborative discussion with HN, RC, and AN ensured that the themes were critically reviewed from multiple perspectives. This collective effort contributed to a comprehensive and balanced data interpretation, further refining the themes and ensuring their robustness.
**6. Writing the report**	MG drafted the initial version of the final report, summarizing the refined themes and analysis. This draft was then reviewed and collaboratively edited by HN, RC, and AN, ensuring that all perspectives were integrated, and the report accurately reflected the findings. The editing helped refine the narrative and conclusions, providing clarity and alignment with the thematic analysis conducted throughout the study.

Table 2. Process during the Deductive Analysis to Address the Second Objective: To What Extent Stakeholders Involved in Developing Maze Out Follow Cahn's Principles of Equality, Diversity, Accessibility, and Reciprocity

Step	Description
**1. Data familiarization**	MG, HN, and DN familiarized themselves with Cahn's principles and then reread the interview transcripts and diaries, noting their initial impressions and observations.
**2. Initial coding**	MG, HN, and DN highlighted passages from the diaries and interviews that they identified as providing information about Cahn's principles.
**3. Developing themes**	MG and HN refined the initial codes and themes through further analysis. Using a deductive approach, MG and HN applied personal and epistemological reflexivity, reflecting on their clinical experience and the underlying assumptions that could influence the interpretation of the data. This reflexive process ensured that the coding and themes remained aligned with Cahn's principles while minimizing the impact of any preconceived notions or biases from the researchers, thereby preserving the integrity of the participants' experiences in the analysis.
**4. Reviewing themes**	In this step, RC, a philosopher specializing in qualitative analysis, joined MG and HN to refine the themes further. Through discussions using critical reflexivity, the team critically examined how their assumptions and biases might influence the analysis. RC's participation provided an external perspective, helping to challenge and deepen the understanding of the themes, ensuring they accurately reflected the data rather than confirming pre-existing expectations. This collaborative process helped to solidify the themes and address potential biases.
**5. Refining and naming themes**	MG developed initial proposals for the refined analysis and then discussed them with HN and RC. AN, with extensive experience in thematic analysis, was also brought in for this step. The collaborative discussion with HN, RC, and AN ensured that the themes were critically reviewed from multiple perspectives. This collective effort contributed to a comprehensive and balanced data interpretation, further refining the themes and ensuring their robustness.
**6. Writing the report**	MG drafted the initial version of the final report, summarizing the refined themes and analysis. This draft was then reviewed and collaboratively edited by HN, RC, and AN, ensuring that all perspectives were integrated and the report accurately reflected the findings. The editing helped refine the narrative and conclusions, providing clarity and alignment with the thematic analysis conducted throughout the study.
