# Supplementary material for: An in-depth understanding of stakeholders’ experiences about their participation in the co-production of ‘Maze Out’: a serious game for the treatment of eating disorders
Source: J Eat Disord. 2024 Nov 14;12:178. doi: 10.1186/s40337-024-01136-3 (PMC11566361; doi:10.1186/s40337-024-01136-3)
Supplement: Supplementary file 3 — Supplementary Material 3 [file 40337_2024_1136_MOESM3_ESM.pdf]

Supplementary File 1: COREQ checklist

Consolidated criteria for reporting qualitative studies (COREQ): 32-item checklist

Developed from:

Tong A, Sainsbury P, Craig J. Consolidated criteria for reporting qualitative research (COREQ): a 32-item checklist for interviews and focus groups. International Journal for Quality in Health Care. 2007. Volume 19, Number 6: pp. 349 – 357

Item No	Description	Reported on Page #
Domain 1: Research team and reflexivity		
Personal Characteristics		
1. Interviewer/ facilitator	MS, a research assistant not otherwise involved in the study, conducted the patient interviews, while MG conducted the interviews with the clinicians and the game designer.	Pg 14
2. Credentials	MD, Psychiatrist, PhD student	Pg 1 and 15
3. Occupation	PhD student	Pg 1
4. Gender	Female	
5. Experience and training	Training as a psychiatrist, psychotherapist, PhD student	Pg 15
Relationship with participants		
6. Relationship established	Yes, with all participants through their practical engagement in the co-production process.	Pg 14
7. Participant knowledge of the interviewer	Participants were aware of MG's role as a clinician working with EDs patients and the reasons for conducting the research.	Pg 14
8. Interviewer characteristics	MS is a final-year public health science student trained in qualitative interviews as part of her studies.<br>MG is a psychiatrist with over 20 years of clinical experience in eating disorders (EDs), and also a psychotherapist. MG employed both personal and epistemological reflexivity throughout the data analysis process. MG's professional background contributed to her potential confirmation bias towards the utility of co-production in treatment development and a psychodynamic theoretical bias	Pg 14,15, 29

Item No	Description	Reported on Page #
	shaped by her clinical practice and psychotherapeutic training. To address these biases, focused discussions with RC, a qualitative analysis philosopher, helped refine interpretations through critical reflexivity.	
Domain 2: study design		
Theoretical framework		
9. Methodological orientation and Theory	Reflexive Thematic Analysis	Pg 2, 25
Participant selection		
10. Sampling	All participants were interviewed.	Pg 14, 28
11. Method of approach	Clinicians face to face, two of the three patients face to face and one by telephone, and a game designer online.	Pg 14
12. Sample size	Two clinicians, three patients, one game designer	Pg 14
13. Non-participation Setting	No participants refused or dropped out.	Pg 14
14. Setting of data collection	Data were collected at the Department of Psychiatry Odense	Pg 14
15. Presence of nonparticipants	No, only the participants and researchers were present during the interviews.	
16. Description of sample	What are the important characteristics of the sample?<br>Appendix 2	Appendix 2
Data collection		
17. Interview guide	MG developed all guides under the supervision of ASN. The interviewees were not informed of the questions in advance. The interviews were used as guides to encourage dialogue on specific topics but allowed flexibility to explore alternative subjects if the interviewees deemed them necessary or important. The interviews were not pre-tested in a pilot project.	Pg 14
18. Repeat interviews	Were repeat interviews carried out? If yes, how many?<br>No	No

Item No	Description	Reported on Page #
19. Audio/visual recording	Did the research use audio or visual recording to collect the data? Audio recordings of interviews- the diaries were analog	Pg 14,15
20. Field notes	MG and MS took notes after the interview. The diaries from patients and one clinician were made during the co-production.	Pg.14
21. Duration	The duration of the interviews was 60 min. approximately.	Pg 14
22. Data saturation	Data saturation was discussed, but it was not a goal of this study, as reflexive thematic analysis was employed. This approach focuses on depth and richness of data rather than achieving saturation, emphasizing the value of meaning-making and flexible interpretation over the collection of data until no new themes emerge	
23. Transcripts returned	Were transcripts returned to participants for comment and/or correction? All participants were allowed to comment on and/or correct their transcripts.	Pg14
Domain 3: analysis and findings		
Data analysis		
24. Number of data coders	Three individuals participated in the familiarization process: MG, HN, and DN. Subsequently, HN and MG completed the coding (details can be found in Appendix 1).	Appendix 1
25. Description of the coding tree	A coding tree was not developed as the analysis process involved iterative discussions and consensus-building around codes and themes, focusing on reflexive thematic analysis rather than formal categorization.	Pg 15
26. Derivation of themes	Were themes identified in advance or derived from the data? Derived from the data in the inductive process.	Pg 15
27. Software	No software was used for data analysis. The coding and analysis were conducted manually through personal discussions among the	Pg 15

Item No	Description	Reported on Page #
	research team, focusing on iterative coding and consensus-building in line with the reflexive thematic analysis approach	
28. Participant checking	Yes, participants were given the opportunity to provide feedback on their transcripts as part of a member-checking process. This allowed participants to comment on and/or correct their transcripts to ensure accuracy and credibility	N/A
Reporting		
29. Quotations presented	Were participant quotations presented to illustrate the themes/findings? Was each quotation identified? e.g., participant number<br>Yes	Pg 17-25
30. Data and findings consistent	Was there consistency between the data presented and the findings?	Pg 17-25
31. Clarity of major themes	Were major themes clearly presented in the findings?	Pg 17-25
32. Clarity of minor themes	Is there a description of diverse cases or a discussion of minor themes?	Pg 25-28
